# Developmental and social deficits and enhanced sensitivity to prenatal chlorpyrifos in PON1^-/-^ mouse pups and adults

**DOI:** 10.1371/journal.pone.0239738

**Published:** 2020-09-25

**Authors:** Ora Kofman, Anat Lan, Eynav Raykin, Ksenija Zega, Claude Brodski

**Affiliations:** 1 Department of Psychology, Ben-Gurion University of the Negev, Be’er Sheva, Israel; 2 Department of Physiology and Cellular Biology, Zlotowski Center for Neuroscience, Faculty of Health Sciences, Ben-Gurion University of the Negev, Be’er Sheva, Israel; Radboud University Medical Centre, NETHERLANDS

## Abstract

The levels and activity of the enzyme paraoxonase 1 affect the vulnerability to the teratogenic effects of organophosphate pesticides. Mutant mice lacking the gene for paraoxonase1 (PON1^-/-^) are more susceptible to the toxic effects of chlorpyrifos, and were hypothesized to be more vulnerable to social behavior deficits induced by exposure to chlorpyrifos during gestation. Three experiments were performed comparing PON1^-/-^ mice to PON1^+/+^ mice born to dams treated with 0.5 mg/kg chlorpyrifos or cornoil vehicle on gestational days 12–15. Chlofpyrifos-exposed male PON1^-/-^ mouse pups had delayed development of reflexes in in the first experiment. In the second experiment, adult male and female PON1^-/-^ mice and the female PON1^+/+^ mice all displayed lower social preference than the male vehicle-treated PON1^+/+^ mice. The PON1^-/-^ mice and the female PON1^+/+^ mice displayed lower social preference compared to the PON1^+/+^ male mice. Male adult mice that had been exposed in utero to chlorpyrifos showed less conditioned social preference regardless of genotype. In the third study, the delayed reflex development was replicated in male and female PON1^-/-^ mice, but chlorpyrifos did not augment this effect. Nest Odor Preference, a test of early social attachment to dam and siblings, was lower in PON1^-/-^ mouse pups compared to PON1^+/+^ pups. This study shows for the first time that PON1^-/-^ mice have a behavioral phenotype that indicates impaired reflex development and social behavior. Chlorpyrifos exposure during gestation tended to augment some of these effects.

## Introduction

Organophosphate pesticides (OPs) are ubiquitous in food, dust, and air. It was estimated that children under age 5 potentially inhale 2 ng/kg/day and potentially ingest close to 5 ng/kg/day, in their home and daycare environments, leading to pervasive exposure to non-toxic levels of CPF [1, 2]. The CHARGE (Childhood Autism Risks from Genetics and Environment) study found a positive association between ASD and residential proximity to OP application during the second and third trimester of gestation in a population-based case control study in California [3]. Landrigan and colleagues [4] stressed the risk for developmental disorders associated with chlorpyrifos exposure in inner city children. More recently, Sagiv et al. [5] found that residential exposure to OPs was associated with poorer scores on social behavior tasks, but was not predictive of autism spectrum disorder (ASD).

Since the liver enzyme paraoxonase is critically involved in metabolizing some organophosphates, such as chlorpyrifos (CPF), paraoxon, and diazonon, [6–8], we hypothesized that variability in function of the gene that controls paraoxonase production (PON1) will critically affect the risk for social behavior anomalies following gestational exposure to CPF. PON1 also confers protection against bacterial endotoxins [9] and other common pesticides whose mechanism of action does not primarily involve acetylcholinesterase (AChE) inhibition [7].

Two common polymorphisms, namely _Q_192_R_, in which glutamine (Q) can be substituted with arginine (R) and _L_55_M_, in which leucine (L) can be substituted for methionine (M), are associated with different levels of enzyme activity. Because variability in PON1 activity is high, even within genotypes [10–12], PON1 status, a measure of both enzyme activity and genotype, is a more reliable index of the protective effects of the enzyme on OP neurotoxicity [13].

Low PON1 activity levels were related to increased severity of OP symptoms in exposed Gulf War veterans [14] and increased OP toxicity in mice [7, 8]. The 192_R_ allele was associated with greater changes in EEG power spectra in the frontal and temporal lobes of people exposed to OP by virtue of residential proximity to an agricultural area [15]. Environmental factors, such as smoking [16–18], diet [19] or alcohol use [11] also modify PON1 activity, thereby influencing the individual’s vulnerability to OP neurotoxicity.

The fetus is critically dependent on maternal PON1 status. Maternal PON1 activity escalates during the course of pregnancy [9, 20], and remains lower in infants than in adults [13, 21, 22].

Studies that suggested that exposure to OP pesticides during pregnancy was associated with symptoms of pervasive developmental disorder (PDD) in toddlers [23, 24] and to autism spectrum disorder (ASD) traits in children [5] raised the question of whether infants born to women with low PON1 status were more susceptible to the detrimental effect of OP exposure during pregnancy. Although an association between autism spectrum disorder (ASD) and the R variant of the PON1 _Q_192_R_ SNP and between the L variant of SNP _L_55_M_, [25] was published, other studies in Europe and in North America did not replicate these findings [26, 27]. A recent study failed to show an association between PON1 levels and neuroimaging markers or IQ in individuals with ASD and low IQ scores [28]. The role of the PON1 genotype as a risk factor for ASD remains unresolved; however, there is accumulating evidence for a link between PON1 genotype or PON1 status and susceptibility to developmental anomalies induced by OP exposure during gestation. Lower birth weight, shorter gestational age and smaller head circumference were reported in newborns, both in Asia and in United States, following *in utero* exposure to OPs in conjunction with low PON1 status in mothers [29–32]. Similar associations were reported in toddlers and children, in longitudinal studies on pesticides. In the Mount Sinai Children’s Environmental Health Cohort study, the Mental Development Index score at 12 months, but not 24 months, was more affected by OP metabolites in black and Hispanic toddlers with the R allele of the _Q_192_R_ polymorphism, but not in those homozygous for the Q allele [32]. On the other hand, PON1 status did not conclusively alter the effect of OP pesticide levels on the frequency of maternal reports of PDD at age two years [33].

When the children were tested at an older age, an interaction between PON1 enzyme activity and maternal levels of OP metabolites was found for scores on the KCPT (Kiddie Connors Continuous Performance Test) at 5 years and scores on the Wechsler Intelligence Scale for Children at 7 years. Specifically, higher paraoxonase levels in maternal blood during gestation were associated with better performance in the child’s perceptual processing speed at ages 5 and 7 years and a lower likelihood to have symptoms of attention deficit hyperactivity disorder (ADHD) at age 5 years [33]. Maternal arylesterase activity during pregnancy modulated the effect of OP pesticide dialkylphosphate (DAP) metabolites on the 7- year old children’s Wechsler Intelligence Scale for Children (WISC) scores, such that the effect of DAP was lower in children whose mothers had high arylesterase activity [33]. The PON1-108_T_ allele was associated with lower scores on the WISC verbal comprehension index at age 7 in association with high levels of OP metabolites in the maternal blood samples from gestation. In the Mount Sinai cohort, the Perceptual Reasoning and Full Scale IQ scores were more negatively affected by maternal exposure to OP pesticides in those children whose mothers who had the PON192_QQ_ genotype. However, no interaction between gestational exposure to OP’s during and PON1 genotypes was found traits related to social skills [34] and non-verbal IQ [35].

Mouse models complement research on behavioral phenotypes that are relevant to the interaction between PON1 activity and the effects of OP exposure. Although there are important limitations to consider [36], preclinical studies enable researchers to deliver a specific dose of OP during a specific developmental period, whereas studies in humans depend on indirect indices of exposure such as levels of metabolites in maternal blood or urine. Many genetic murine models [37–39] have been tested on validated behavior tests, making the mouse suitable for investigating the interaction between PON1 genotype and OP exposure on assays of ASD.

Prenatal exposure to CPF on gestational days (GD) 12–15 in mice delayed the development of sensorimotor reflexes, increased repetitive behavior, impaired social preference, and also impaired conditioned place preference for both a social and a non-social cue [40, 41]. Mutant mice lacking the PON1 gene (PON1^-/-^) are more vulnerable to the toxic effects of several AChE inhibitor pesticides [8, 41]. However, it is not known if these mutant mice are also susceptible to the subtle long-term social behavioral effects of gestational exposure to CPF. Exposure to the active oxon metabolite of CPF, chlorpyrifos oxon (CPO), administered on postnatal days 4–21 in PON1^-/-^ mice led to a significantly delayed latency to reach maximum startle amplitude. On the other hand, the development of three neonatal reflexes and spatial and fear learning were unimpaired in the adult PON1^-/-^ mice exposed to CPO [41]. Nevertheless, the exposure to CPO on days 4–21 significantly disrupted the expression of several genes related to cell and synaptic function [42]. Similarly, CPF had long-term effects of the expression of genes involved in nervous system development even in doses that show no overt signs of OP toxicity, [43, 44].

We hypothesized that PON1^-/-^ mice might be more susceptible than the C57Bl/6 strain to the effects of prenatal exposure to CPF on neurodevelopment and social behavior. In the first 2 experiments we compared the behavior of C57Bl/6 (B6) mice, the recommended comparison genotype for this mutant strain (https://www.jax.org/strain/004160) with PON1^-/-^ mice born to dams treated with 0.5 mg/kg CPF or corn oil and after the mutant mice were commercially available, we purchased (Jackson Laboratory, Bar Harbor ME) and bred PON1^-/-^ and PON1^+/+^ mice.

The dose used for the PON1 ^-/-^ mice was 10% of the highest dose used in our previous studies on B6 mice. This dose did not elicit any overt signs of cholinergic toxicity as shown in our studies and those of other groups [40, 44–47]. By comparison, rodent dams and fetuses have been found to tolerate repeated doses of 25 mg/kg [48] or even 40 mg/kg CPF [49] without overt toxicity.

We hypothesized that a low dose of CPF (0.5 mg/kg) administered to dams on GD 12–15 in PON1^-/-^ mice would delay the development of sensorimotor reflexes, disrupt nest odor preference (NOP) in pups and social preference in adult offspring. In order to test social behavior in pups and adults, PON1^-/-^ and PON^+/+^ mice were tested following exposure to CPF on gestational days 12–15, corresponding to Theiler stages 23–26 [50] and days 61–84 in human cerebral cortex and days 50–67 in human limbic structure development [51].

## Materials and methods

### Ethics statement

All procedures were approved by the Ben-Gurion University of the Negev University Committee for the Care and Use of Animals in Experiments (Protocol: IL 55-08-15). Mice were housed in an AALAC-approved facility (Association for Assessment and Accreditation of Laboratory Animal Care) with controlled temperature (21-23^o^ C) and a reverse 12 hr light-dark cycle, so that testing occurred in the dark phase. Following completion of each study, mice were euthanized by the facility’s trained personnel with CO2.

### Mouse breeding

Male and female B6 (referred to herein as PON1^+/+^) mice were purchased from Harlan, Israel and served as the control mice. Mutant PON1^-/-^ mice were originally developed from 129/SvJ mouse embryonic stem cells, and then crossed back to a B6 background [8]. For Experiments 1 and 2, mice were bred from male and female PON1^-/-^ generously donated by Prof. Michael Aviram in the Technion, Israel, with the generous agreement of Dr. Shih. Mice in Experiment 3 were purchased from Jackson Labs, as PON1^-/-^ and PON1^+/+^ mice which were at that time commercially available.

To breed mice, one male was housed with two females that were checked daily for the presence of a copulatory plug within 30 min of the start of the dark phase of the cycle. Upon detection of the plug, the female mouse was weighed, housed separately and the date was recorded as Gestational Day zero (GD 0). The day of birth was designated postnatal day (PND) 0. On PND 28, the pups were weaned and housed with same sex littermates. Genotype was confirmed by PCR using a tail snip from the dams and sires before breeding as previously described [52].

Three experiments were conducted, as follows:

*Experiment 1*: Reflexes were tested in male pups only in the initial study to reduce the number of mice. Only in subsequent experiments were both sexes tested to replicate the results and extend them to females.

*Experiment 2*: Male and female adult mice were tested for spontaneous and learned social preference (social preference (SP), social conditioned place preference (SCPP), respectively) and in a control task, food conditioned place preference (FCPP).

*Experiment 3*: Reflexes were tested in male and female pups and the Nest Odor Preference (NOP) task, a test of social behavior in pups, was tested in naïve pups.

In all experiments only one male and one female pup per litter were tested: 41 litters in Experiments 1 and 2 and 31 litters in Experiment 3.

### Prenatal chlorpyrifos administration

Chlorpyrifos (99.5% purity ChemService Inc.) (CPF), was suspended in corn oil in a concentration of 0.5 mg/10 ml. CPF or corn oil vehicle (Willi Food, Yavneh, Israel) was administered by oral gavage to the pregnant dam in a volume of 10 ml/ kg body weight on GD 12–15. The dose was one tenth of the dose that found to delay the development of reflexes in pups and to impair social preference and conditioned social place preference in adult PON1^+/+^ mice in our lab [40, 45]. This dose was used in this study as PON1^-/-^ mice are more susceptible to the toxic effects of organophosphate pesticide [8]. All care and handling during the breeding period was carried out by the experimenters.

### Behavioral testing

Reflexes in pups were tested as previously described [40, 45, 53, 54]. The pups were weighed before testing and weight used as a covariate in the analysis of variance to control the possible difference in body size in the development of reflexes. Social behavior in pups was assessed using the NOP test [55, 56]. Preference for maternal odor over that of unsoiled bedding or bedding from a different litter’s nest was deficient in mice with a diminished emotional response to dams [57].

The Social Preference (SP) test, validated in a variety of genetic mouse models of ASD [38, 39], assesses the extent to which the mouse prefers the presence of a conspecific over that of an inanimate object. Conditioned place preference is a Pavlovian learning method widely used in the study of drug addiction [58] and brain stimulation reward [59]. Social conditioned place preference (SCPP) utilizes this paradigm to demonstrate that cues associated with social vs isolated housing conditions are rewarding [60]. In order to test the specificity of the social behavior tests, we also introduced a control non-social conditioning test, namely food conditioned place preference (FCPP) to address the question of whether SCPP deficit involves a general learning deficit.

#### Experiment 1: Sensorimotor reflexes on PND 5–12 in males

On postnatal days (PND’s) 5–12 three neurodevelopmental reflexes were assessed. The *righting reflex* was tested by placing the pup on its back and timing the latency to turn over to an upright position. *Negative geotaxis (NG)* was tested by placing the pup facing downward on a metal screen grid at a 30^o^ incline. The ability of the mouse to reorient itself from a head down position was observed for 30 seconds. *Cliff avoidance (CA)* was tested by placing the pup on the edge of a flat surface so that its whiskers could not contact any surface. The mouse was observed for up to 30 seconds and scored for its ability to avoid the cliff by backing away or turning around. NG and CA were scored as follows: 1 –falling (CA) or slipping downwards (NG); 2- remaining in the same position or 3- turning at least 90^o^ from the edge for CA or making a 180^o^ turn to orient the head upwards for NG. Each pup was tested three times consecutively and the daily score was the average of three trials. If the reflex was not performed within 30 sec, the trial was scored as a failure (30 for righting reflex and score 1 for CA and NG).

Pups born to dams whose plugs were not detected and therefore could not be treated precisely on GD 12–15, were used as a non-treated control group in Experiment 1, to control for the stress of gavage during pregnancy. In Experiments 2 and 3 untreated mice were bred to serve as stimulus mice in the nest odor preference and social preference experiments. In all cases the pups were born to dams that had been purchased and bred at the same time as the experimental mice so that there was no difference between the groups except for the treatment by gavage.

#### Experiment 2. Social preference and conditioned social preference in adult male and female PON1^-/-^ mice exposed to CPF on GD 12–15

Social preference was conducted in naïve adult (4–5 months) mice from the same litters used in Experiment 1 at age 4–5 months as described in Lan et al. [40, 45]. The SP apparatus consisted of a clear Perspex rectangular box (60 x 40.5 x 25.5 cm), that had a removable washable white plastic posterboard floor to facilitate detection of the mouse by the Ethovision tracking program. The box was divided into three equally sized chambers interconnected by small openings to allow passage of the mouse between chambers. A day before testing, the experimental mouse was placed in the apparatus for 10 minutes to explore and become habituated to box in the absence of the social or non-social stimuli. The stimulus mice were habituated for 10 minutes to the enclosed container one day prior to the test.

On the test day an overturned perforated opaque plastic container (0.4 X 9.4 cm x 12.2 cm high) was placed in each side chamber. Under one container an untreated stimulus mouse of the same sex and age was placed to act as the social stimulus. The container on the opposite side was empty and served as the object (non-social) stimulus. The sides were counterbalanced within each treatment group. The mouse was placed in the middle chamber with passages blocked by removable barriers. The session began as the experimenter lifted the barriers. The session was filmed from above and coded off-line using the Noldus Ethovision® system.

In order to score preference, the social and object chamber were each sub-divided into a proximal and distal zone. The proximal zone was formed by drawing a rim of 3 cm around the circumference of the container. The remainder of the mouse-containing zone was labelled as the distal social zone. A similar configuration was used to delineate proximal and distal object (non-social) zones. Time spent in each zone was measured and then was divided by the total time spent in all four zones, yielding four measures: social-proximal (SP) social-distal (SD), object-proximal (OP) and object-distal (OD).

*Social conditioned place preference (SCPP)* was conducted in a separate cohort of mice at PND 90 using the same apparatus that was used in the SP assay. First, the mice were given the choice of choosing between two novel beddings, aspen sawdust or shredded paper, and their preference was recorded for 10 minutes, by scoring how much time was spent on the side of each of the bedding materials. Each mouse received a preference score (Preferred bedding / Total time with both beddings). The conditioning phase began by placing the mouse with a same-sex cagemate from the same litter in a cage lined with the non-preferred bedding on 5 alternate days. On the other days, the experimental mouse was alone in a cage lined with the initially preferred bedding. Thus, the initially preferred bedding was paired with isolation and the initially non-preferred bedding was paired with social living conditions in order to alter the preference through social conditioning. The mice had *ad lib* food and water.

The conditioned preference for the two distinct types of bedding was assessed in the three-chambered apparatus for 10 minutes where the floor of each side was covered with one of the bedding materials. The sides on which the social or isolation bedding was placed were counter-balanced between animals to control for a possible spatial bias. Time spent in each chamber was scored via Ethovision software by an observer blind to the treatment condition. The preference score was calculated as above. The change in preference was calculated as the post-conditioning minus the pre-conditioning score. A positive score indicates that the bedding preference changed following the social conditioning.

*Food conditioned place preference (FCPP)* Failure to show conditioned social place preference is commonly claimed to be evidence for a social deficit [61]; however, it is plausible that mice have a more general deficit in classical conditioning. In order to test if impaired conditioning was generalized or was indeed restricted to social cues, a control Food Conditioned Place Preference (FCPP) experiment was conducted in a similar manner, using a food reward instead of a social reward. A separate cohort of mice was pretested for their initial bedding preference and then housed alone on alternate days with a food reward or with no food reward. During this period, regular chow was limited to a portion limited to 9% of body weight in grams (i.e. a mouse weighing 30 gr received 2.7 gr chow on that day). In order to associate the bedding with a food reward, 10 soup croutons (Osem ®) were buried in the initially non-preferred bedding so that the mouse was required to forage in the bedding to obtain this snack. On alternate days, the mouse was housed with the initially preferred bedding with no extra food. After alternating treatment for 10 days, the bedding preference was tested and analyzed as described for the SCPP.

#### Experiment 3: Reflex development and social attachment in male and female PON1^-/-^ mice and B6 mice following prenatal CPF exposure on GD12-15

*Sensorimotor reflexes*: Mice were bred from PON1^-/-^ and PON1^+/+^ mice purchased from Jackson Ltd and genotyped before breeding by real-time PCR of tail samples. Treatment was done as described in Experiment 1. One male and one female per litter was tested on postnatal days 2, 4, 6, 8, and 10 with Day as a repeated measure. The tested pup was marked by a tail snip on the day of birth for identification. As described above, each daily score was the average of three consecutive trials and the pup was placed on a warm surface between trials. Failure to complete the reflex response within 30 sec was scored as a failure. Scoring was performed as in Experiment 1.

**Fig 1:**
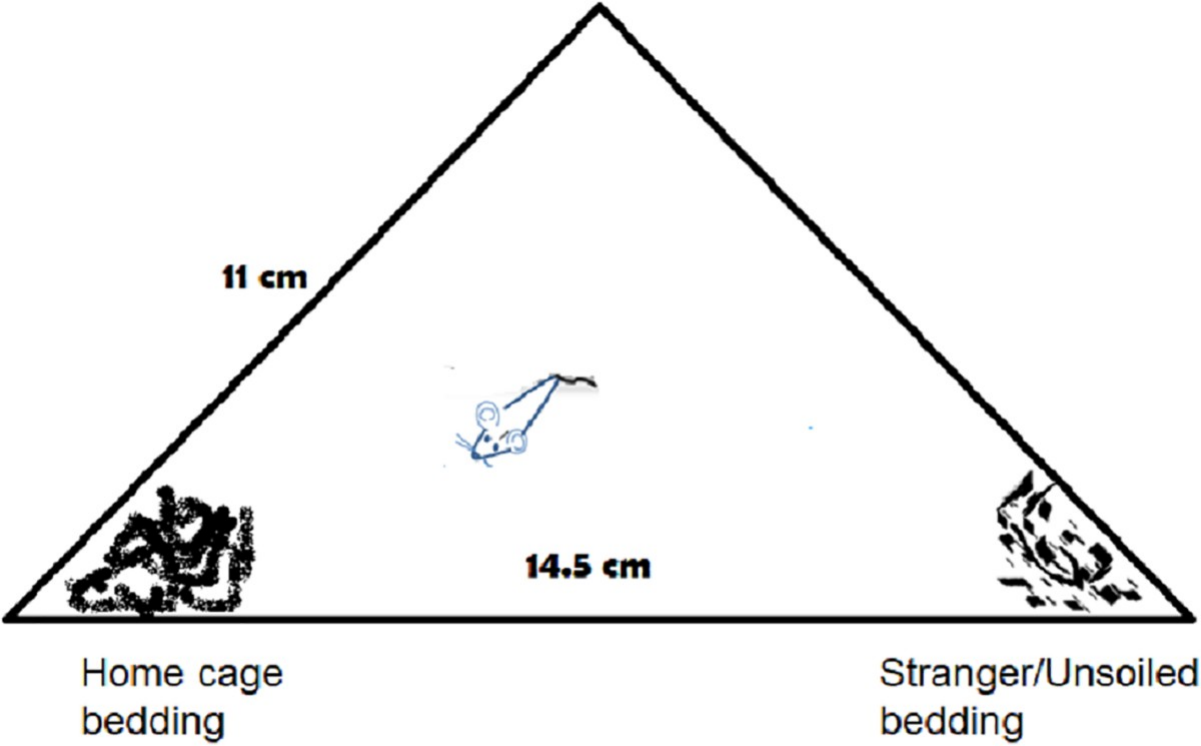
Schematic drawing of the apparatus used to measure nest odor preference in mouse pups in Experiment 3.

*Nest Odor Preference (NOP)*: NOP was tested on one naïve male and one naïve female from the same litters that were used for the reflex testing. NOP was tested on PNDs 8, 10 and 12, for 180 sec. The pup was placed in the middle of a triangular plastic enclosure, 14.5 x 11 x 11 cm (Fig 1). Two tablespoons of bedding were placed in each corner so that the pup could explore and sniff each area. Two conditions were tested for preference of the soiled home cage bedding over the contrasting stimulus bedding. In the UNSOILED condition fresh sawdust was placed in one corner and soiled bedding from the home cage (HOME) was placed in the opposite corner. In the STRANGER condition, soiled bedding from another litter, born to a vehicle-treated dam, was placed in one corner and soiled litter from the home cage was placed in the opposite corner. The pup’s movements were filmed and scored off-line for the time spent with each of the beddings by an observer blind to the treatment group. The side on which the home and test beddings (UNSOILED or STRANGER cage) were placed and the order of testing were counterbalanced. The pup was placed back in its home cage with the dam and litter for 5 minutes between each trial. Nest Odor Preference (NOP) was calculated as [(Time spent in home bedding minus time with test bedding)/ 180 sec], such that positive scores reflected preference for the home cage odor.

### Statistical analysis

Each experiment was analyzed by ANOVA using the Statistica program, version 12 ®StatSoft, Inc. [62]. 3-way ANOVA was used for the effect of Genotype x Treatment X Day as a repeated measure for Experiment 1 with males only. Weight was used as a repeated covariate for the analysis of pup reflexes. For Experiments 2 and 3, in which males and females were tested, a 4-way ANOVA was used for analyzing reflexes and NOP (Genotype X Treatment X Sex X Day and a 3-way ANOVA for the effect of Genotype x Treatment X Sex for the SP, SCPP and FCPP tests. Interactions were analyzed using the Scheffé or Duncan post hoc test for the hypothesized effects. The Levene’s test for homogeneity of variance was applied and if it was significant, the relevant effect was analyzed by a non-parametic Kruskal Wallis or Chi-square tests.

## Results

### Experiment 1: Sensorimotor reflexes on PND 5–12

#### Weight

**Fig 2:**
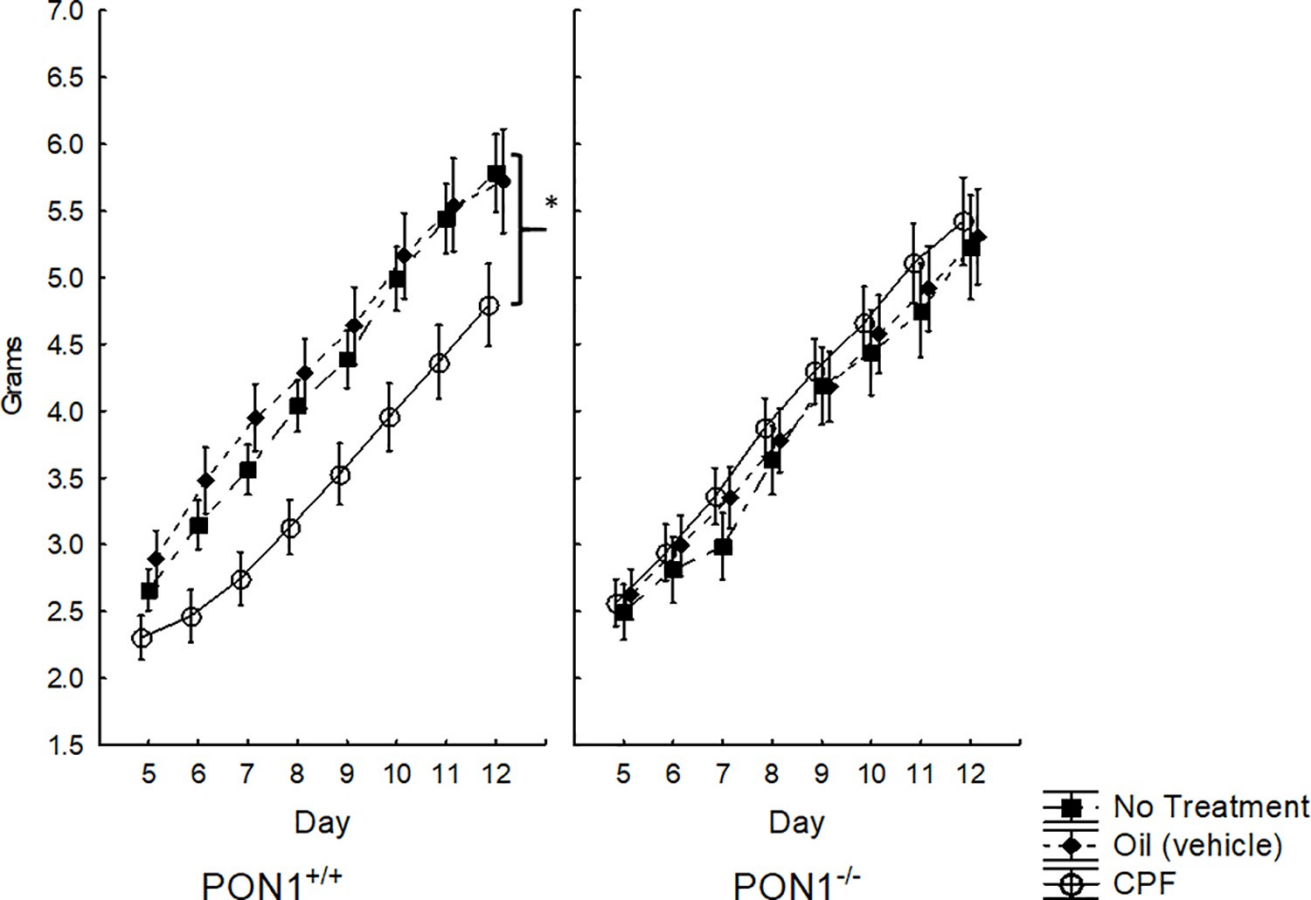
Mean + SEM body weight in grams for mouse pups, showing the significant interaction between Genotype and CPF. PON1^+/+^, but not PON1^-/-^ mice, exposed to CPF had lower body weight. * CPF vs vehicle (p < .001), CPF vs NT (p < .03). Number of animals per group as follows: B6-NT n = 9, B6-Vehicle n = 5, B6-CPF n = 8. PON1^-/—^NT n = 5, PON1-/-—Vehicle n = 6, PON1-/-—CPF n = 7.

A three-way ANOVA with repeated measures for the effect of Day (PND 5–12) as a repeated measure, Treatment (NT-No Treatment, Vehicle, 0.5 mg/kg CPF) and Genotype (PON1^+/+^, PON1^-/-^) on body weight revealed the expected significant effect of Day indicating weight gain with age, F (7, 238) = 429.08, p < .0001. In addition, a significant Genotype X Treatment interaction was found, F (2, 34) = 3.74, p = 0.034. Duncan’s post hoc comparisons revealed that in PON1^+/+^ mice, the gestational exposure to the low dose of 0.5 mg/kg CPF resulted in lower weight gain compared to the two control group: vehicle (p < .001), and no treatment (NT) (p < .03) (Fig 2). Since there were significant differences in the rate of weight gain, weight was used as a covariate for the reflex ANOVA.

#### Righting reflex

**Fig 3:**
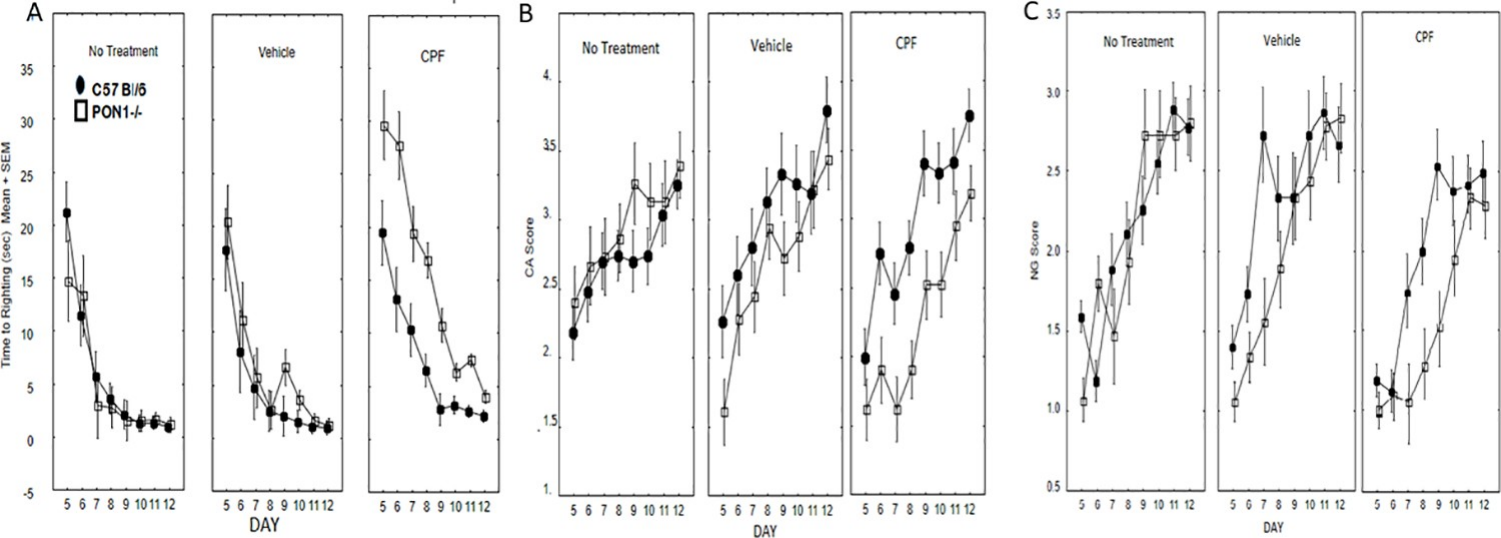
Mean + SEM scores of neonatal reflexes. A: Righting reflex, B: Cliff Avoidance and C: Negative Geotaxis. N for each group as in Fig 2.

A three-way ANOVA for the effects of Treatment X Genotype X Day (repeated measure) with daily body weight as a covariate was conducted on the righting reflex score. The expected effect of Day was significant F (7, 238) = 70.39, p < .00001, indicating more rapid performance of the righting reflex as the mice matured. There was a significant main effect of Treatment, F (2, 34) = 16.30, p < .0001 and a significant Day X Treatment interaction F(14, 238) = 2.29, p = .006. There was also a main effect of Genotype, F (1, 34) = 7.87, p = 0.008, modified by a Treatment x Genotype interaction, F(2,34) = 7.28, p = .002 (Fig 3A). Duncan’s post hoc tests conducted on the latter interaction confirmed that the PON1^-/-^ mice that had been exposed to CPF had delayed development of the righting reflex compared to the other groups. The Levene’s test for homogeneity of variance was significant for each day except postnatal days (5–6 p<0.01). A non-parametric rank comparison using the Kruskal-Wallis test confirmed that on each test day except PND 5 (p = 0.06) there was a significant effect of group in performance of the righting reflex) PND 6 p = 0.03; PND 7–8 p = 0.003; PND 9 p = 0.01, PND 10 p = 0.002; PND 11 p = 0.0009); PND12 p = 0.01).

#### Cliff avoidance (CA)

The three-way ANOVA for the effects of Treatment X Genotype X Day (repeated measure), with daily body weight as a covariate revealed the expected significant effect of Day, F(7, 238) = 27.94, p < .00001, reflecting improvement with age. There was also a main effect of Genotype F (1, 34) = 6.43, p = 0.016, which was modified by a significant Genotype X Treatment interaction, F(2,34) = 6.36, p = .004. Duncan’s post hoc comparisons for this interaction revealed that the PON1-/- mice that had been exposed prenatally to CPF performed the CA reflex more poorly than each of the other groups (p < .04 for all comparisons) (Fig 3B) throughout the testing period. The Levene’s test for homogeneity of variance was not significant for CA.

#### Negative geotaxis

The three-way ANOVA for the effects of Treatment X Day X Genotype (repeated measure), with daily body weight as a covariate, revealed the predicted improvement with age as seen by the main effect of Day, F (7, 238) = 54.82, p < .0001. There were main effects of Genotype, F (1, 34) = 5.86, p = .021 and Treatment, F (2,34) = 7.23, p = .002, that were modulated by a 3-way interaction between Day x Genotype x Treatment, F (14, 238) = 1.77, p = 0.043. Duncan’s post hoc comparisons revealed that PON1^-/-^ mice that were treated with CPF were slower to develop the NG reflex compared to all of the other groups (p < .03 for all comparisons). Notably, on PND 11 and 12 there were no differences between the groups (p>.05), indicating that the impaired NG reflex in the PON^-/-^ mice whose dams had been gavaged with 0.5 mg/kg CPF recovered well before they were weaned on PND 21 (Fig 3C). Levene’s test for homogeneity of variance was significant on Days 5 (p = 0.00001) and 7 (p = 0.01). Therefore, the Kruskal Wallis test was conducted confirming that the PON1-/- groups had a lower reflex score on these days (p<0.01 on each day).

### Experiment 2. Social preference and conditioned social preference in adult male PON1^-/-^ mice exposed to CPF on GD 12–15

#### Social preference (SP)

A four-way ANOVA for the effects of Genotype, Sex, Treatment and Zone as a repeated measure was conducted for the time spent in each zone divided by the total time in all four zones during the test period. The four zones were designated as social-proximal (SP), social- distal (SD), object-proximal (OP) and object-distal (OD). The Levene’s tests for homogeneity of variance were insignificant with F values <0.

**Fig 4:**
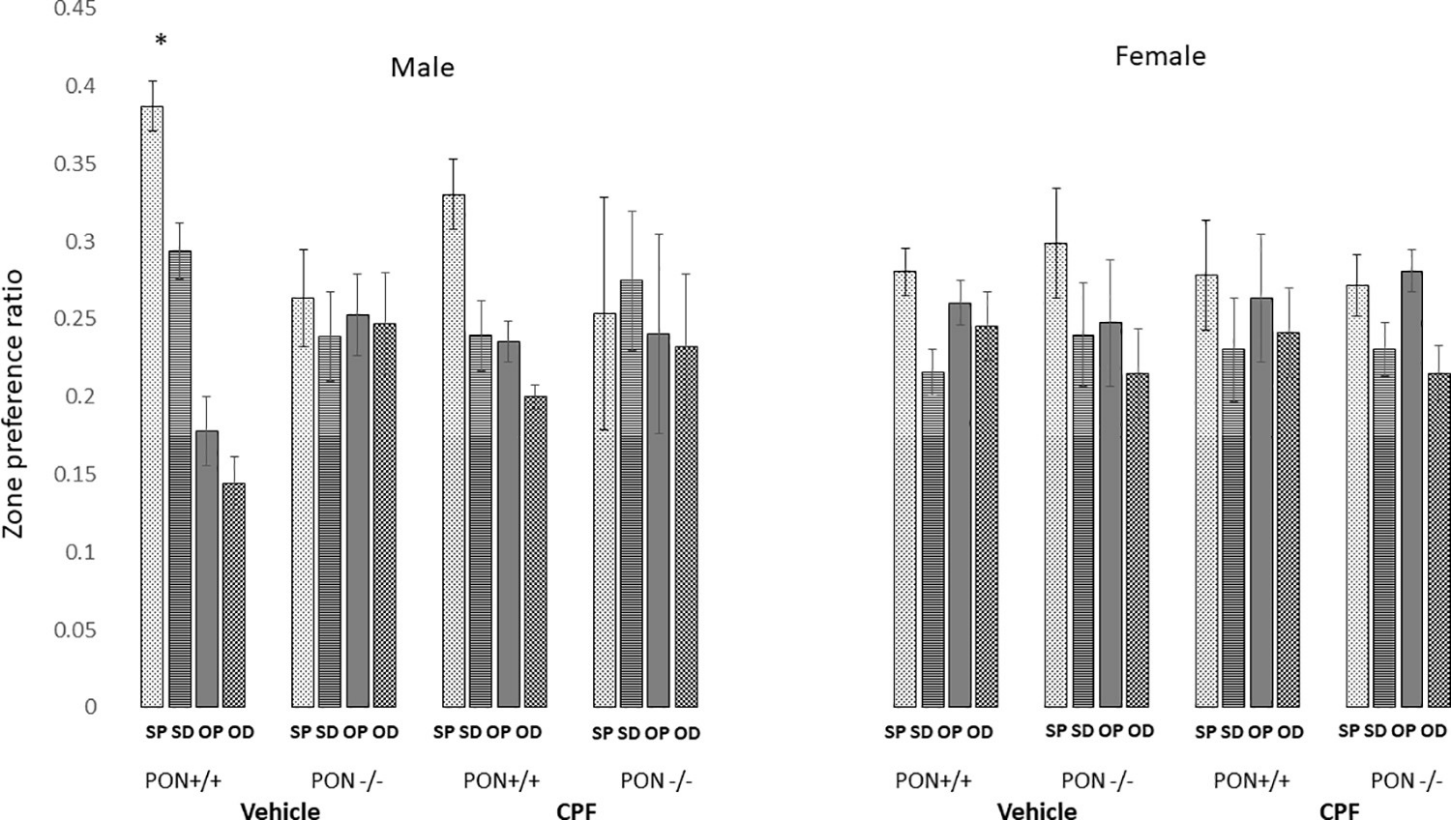
Mean + SEM preference for another conspecific. * p<0.005 compared to each of the other zones. The number of mice per groups is in parentheses as follows: PON1^+/+^ Males vehicle (10), males CPF (5), Females vehicle (10), females CPF (8), PON1^-/-^ Males vehicle (6), males CPF (5), Females vehicle (4), females CPF (6).

A main effect of Zone was found as there was the predicted preference for the zone closest to the stimulus mouse, zone SP, F (3,138) = 8.83, p < .0001. This was modulated by a significant Zone X Genotype X Sex interaction, F (3,138) = 4.33, p = .006. Post-hoc Duncan’s analysis of this interaction showed that the PON1^+/+^ male mice spent significantly more time in the SP zone, compared to each of the other groups. As can be seen in Fig 4, female B6 and female and male PON1^-/-^ mice did not show a preference for the zone proximal to the stimulus mouse. The failure of the male PON1^-/-^ mice to acquire SP, suggests an inherent deficit in social preference.

#### Social conditioned place preference (SCPP)

**Fig 5:**
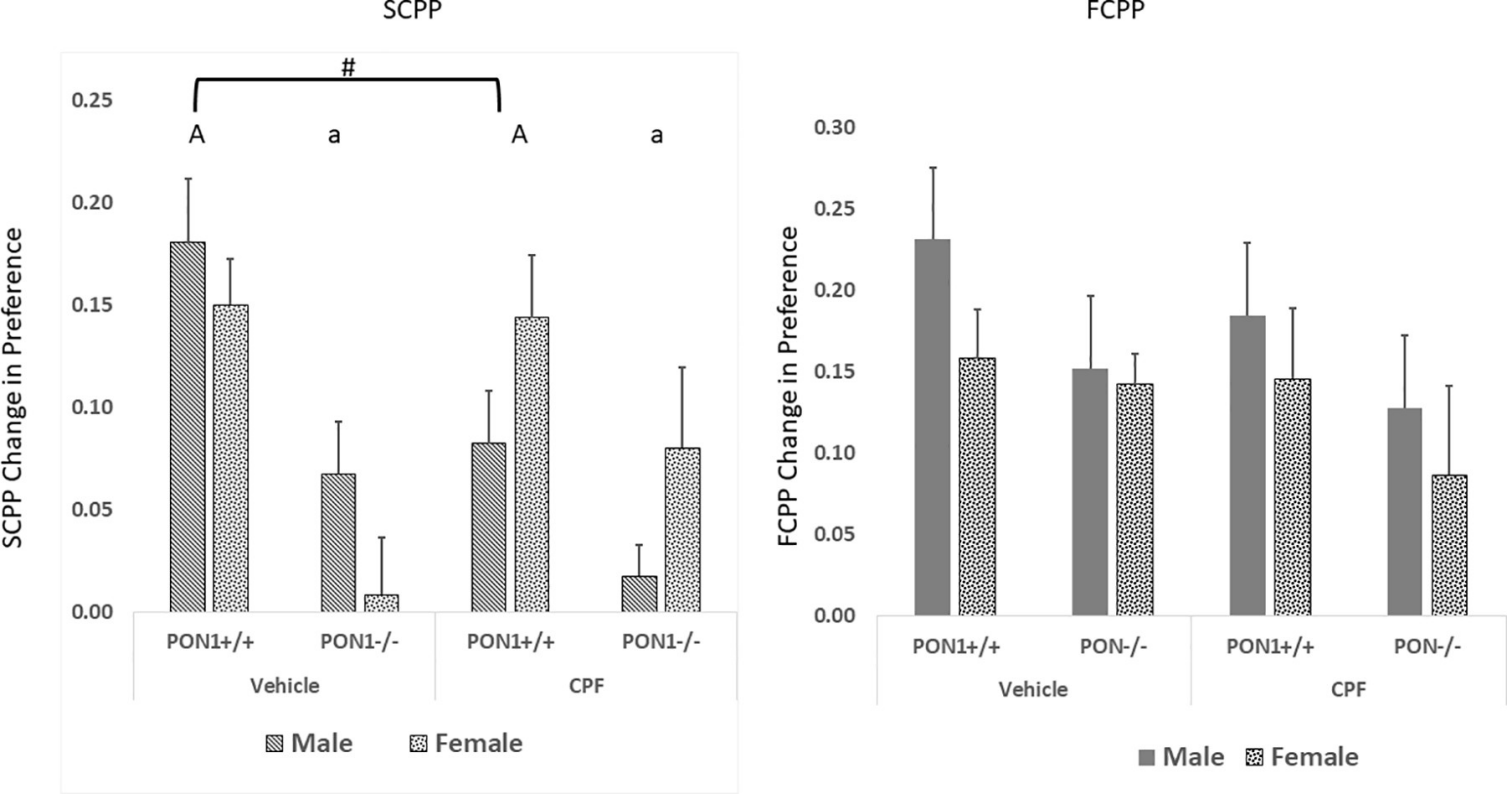
Left panel SCCP. Mean + SEM change in preference for the bedding conditioned to a social stimulus. A vs a. Main effect of genotype. #—Main effect of Sex. Number of mice in parentheses for each group: Male PON1^+/+^ Vehicle (5); CPF (4); Female PON1^+/+^ Vehicle (4); CPF (3); Male PON1 ^-/-^ Vehicle (6); CPF (5); Female PON1^-/-^ Vehicle (5) CPF (4) Right panel FCPP. Mean + SEM change in preference for the bedding that was conditioned to a food stimulus. Number of mice/group: Male PON1^+/+^ Vehicle (10); CPF (5); Female PON1^+/+^ Vehicle (10); CPF (8); Male PON1 ^-/-^ Vehicle (6); CPF (5); Female PON1^-/-^ Vehicle (4); CPF (6).

In the SCPP task, the change in preference, calculated as the preference ratio in the post-conditioning test minus the pre-conditioning preference ratio was analyzed by a 3-way ANOVA for the effects of Genotype, Treatment and Sex. Levene’s test for homogeneity of variance was not significant, F = 1.67, p = 1.29. A significant main effect for Genotype was found, F(1,72) = 18.34, p < .00001, indicating that PON1^-/-^mice showed decreased SCPP compared to PON1^+/+^ mice. In addition, a significant Sex X Treatment interaction was found, F (1, 72 = 5.72), p = 0.019. Duncan’s post hoc test revealed that the SCPP of males whose dams had received 0.5 mg/kg of CPF during gestation was lower than that of the males whose dams had been gavaged with the oil vehicle. That difference was close to conventional statistical significance (p = .054) and not related to genotype. No effect of CPF on SCPP was found in the female mice (Fig 5A).

#### Food conditioned place preference (FCPP)

The ratio of time spent with the initially non-preferred bedding prior to and following food conditioning was analyzed by a 3-way ANOVA for the effects of Genotype, Treatment and Sex. Levene’s test for homogeneity of variance was not significant, F = 0.60, p = 0.75. No main effects or interactions were significant in this test; however, the main effect of Genotype was close to significant, demonstrating that the PON1 ^-/-^ were less likely to undergo classical conditioning associating even in a non-social setting F(1,32) = 4.03, p = 0.053), (Fig 5B).

### Experiment 3: Reflex development and social attachment in male and female PON1^-/-^ mice and B6 mice following prenatal CPF exposure on GD12-15

#### Weight

**Fig 6:**
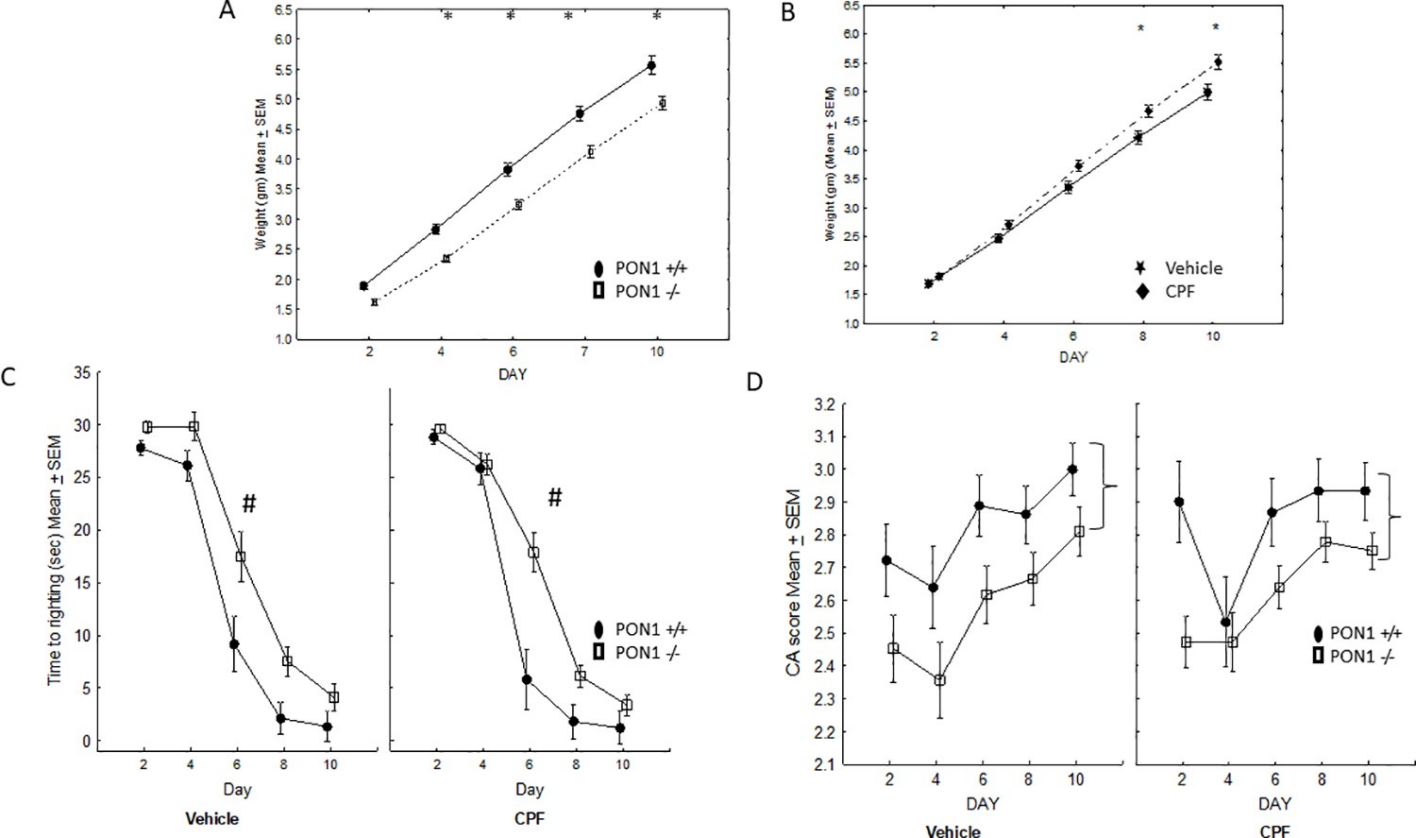
A. Mean + SEM body weight of mouse pups in Experiment 2 showing Genotype x Day interaction. PON1^-/-^ had lower weight (p<0.005) on all days except day 2 (p = 0.056). B. Effect of prenatal exposure to CPF on body weight. * p<0.05. C. Time until completion of righting reflex. # Day 6 PON1 ^-/-^ were significantly slower than PON1^+/+^ mice, p<0.00001. D. Cliff Avoidance reflex score.} indicates main effect of genotype showing lower scores in the PON1^-/-^ mice. The number of mouse pups per group is: PON1^+/+^–Vehicle = 6 M and 6 F; PON1^+/+^—CPF = 5 M and 5 F. PON1^-/-^ Vehicle = 7 M and 7 F; PON1^-/-^ CPF = 12 M and 12 F.

A four-way ANOVA for the effect of Genotype (PON^-/-^ vs PON1^+**/+**^), Sex (M, F), Treatment (Vehicle vs 0.1 mg/kg CPF) with Day (2, 4, 6, 8, 10) as a repeated measure showed a significant effect of Genotype, F(1, 52) = 18.44, p < .0001, and of Treatment, F(1, 52) = 7.91, p < .01 and the expected significant increase of weight across days, F(4, 208) = 1646, p < .000001. There was also a significant interaction between Genotype and Day F (1, 52) = 4.88, p < .001. A post-hoc Duncan test showed that the PON1^-/-^ mice had significantly lower body weight than the PON1^**+/+**^ mice on each of the test days, p<0.005, except on Day 2 where the difference was not significant, p = 0.056 (Fig 6A). The significant interaction between Treatment and Day F(1, 52) = 5.56, p < .001, was further analyzed by a post-hoc Duncan test. On Days 8 and 10 the pups born to CPF-treated dams weighed more than those born to vehicle treated dams (Fig 6B). Daily weight was used as a covariant for the analysis in the ANOVA that tested for differences related to Genotype, Treatment and Sex, for each of the three reflexes, using Day as a repeated measure

#### Righting reflex

Performance of the righting reflex improved with age, as seen by the significant effect of Day F (4, 208) = 322.60, p< 0.00001. There was a significant effect of Genotype, F(1,52) = 20.10, p < .0001, modified by a Day x Genotype interaction F(4, 208) = 6.82, p < .0001. A post-hoc Scheffé test indicated that the PON1^-/-^ mice had slower righting on PND 6 (p < .000001). There were no interactions and no main effect involving CPF treatment, suggesting that the PON1^-/-^ genotype is associated with delayed development of the righting reflex, but that this difference was transient (Fig 6C). Levene’s test for homogeneity of variance was significant. The effect of treatment and genotype was analyzed by the non-parametric Kruskal Wallis test for each test day. On PNDs 6 amd 7 both the vehicle and CPF-treated PON1 -/- groups had significantly slower righting reflexes (p = 0.008 and p = 0.002, respectively), with no effect of CPF treatment.

#### Negative geotaxis

The negative geotaxis reflex improved with maturation as shown by the main effect of Day, F (4, 208) = 58.50, p< 0.000001 and there was a significant interaction between Treatment and Sex in the execution of this reflex, F (1,52) = 4.61 p = 0.036. Females who had been pre-exposed to CPF during gestation performed this reflex better than males (p<0.05, post-hoc Duncan test), regardless of Genotype (data not shown).

#### Cliff avoidance

Cliff avoidance improved with maturation as shown by the main effect of Day F (4, 108) = 10.45, p< 0.000001. In addition, there was a main effect of Genotype, indicating poorer performance of the PON1 ^-/-^ mice, F (1, 52) = 21.80 p< 0.001. There were no interactions and no main effect involving CPF treatment, (Fig 6D).

#### Nest odor preference

**Fig 7:**
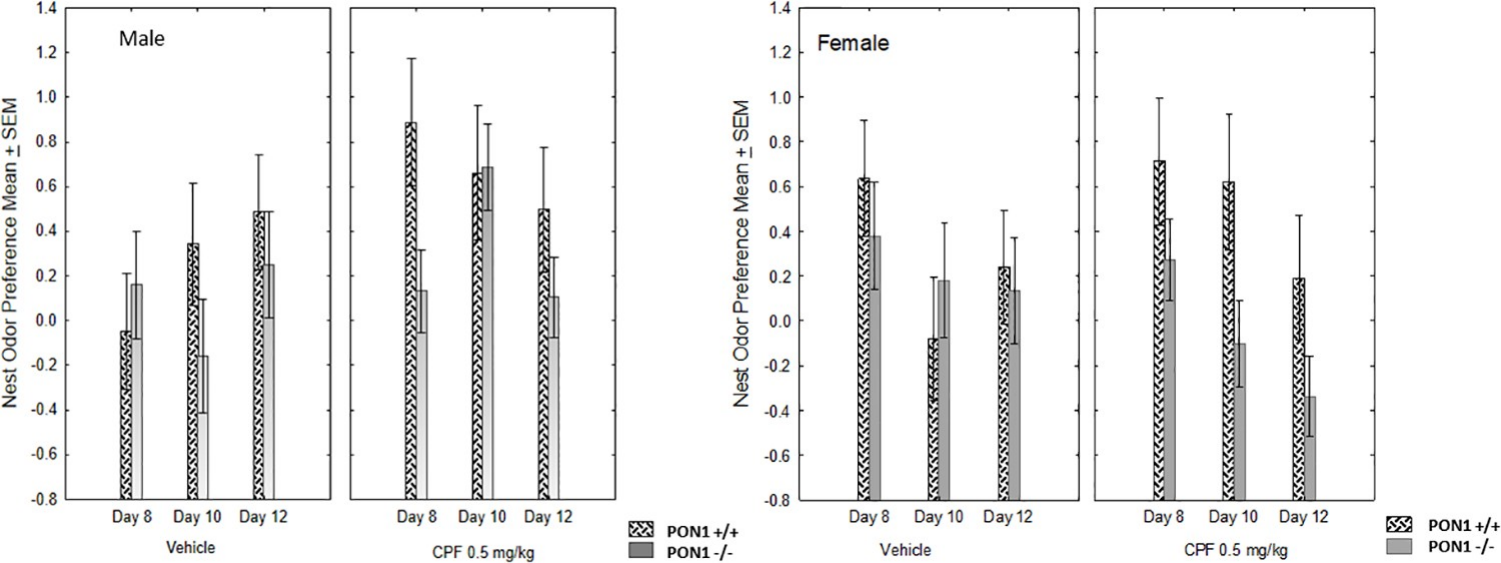
Nest Odor Preference (NOP) score (Mean + SEM) compared to unsoiled bedding in PON1^+/+^ and PON1^-/-^ mice born to dams treated on GD12-15 with either oil (vehicle) or CPF. Pups were tested on postnatal days 8, 10 and 12. NOP was lower in PON1^-/-^ mice (p < .005) compared to PON1^+/+^ mice. PON1^-/-^ mice exposed on GD 12–15 to CPF had a lower NOP score than PON1^+/+^ mice exposed prenatally to CPF (p<0.05). The number of pups per group is given in the legend to Fig 4; however, in this experiment naïve pups from the same litter were tested.

A -way ANOVA for the effects of Genotype X Treatment X Sex X Day, with Day as a repeated measure (day 8, 10 and 12) was calculated for NOP for the Unsoiled and Stranger conditions. All groups showed a positive value, indicating that there was an overall preference for the home nest odor. Notably, NOP in the Unsoiled condition was lower in the PON1^-/-^ mice compared to the PON1^+/+^ mice, F (1,52) = 9.11 p = 0.004. Since the Genotype x Treatment interaction was near significant according to our *a priori* hypothesis, F (1,52) = 1.34 p = 0.060, a post-hoc analysis of this interaction was conducted to understand the effect of prenatal CPF. The Duncan post-hoc test showed that PON1^+/+^ mice pre-exposed to CPF on GD 12–15 had a higher NOP than the PON1^-/-^ mice exposed to CPF (Fig 7). This confirmed that PON1^-/-^ mice are more sensitive to impaired social behavior induced by prenatal exposure to CPF. According to the Levene’s test for homogeneity of variance, for genotype, the NOP data were not normally distributed for day 12. Therefore, we also analyzed these data using the χ^2^ test for the effect of Genotype for each day separately. This test showed that the PON1^-/-^ mice had lower NOP than the PON1^+/+^ mice on PND 12, χ^2^ = 4.59, p = 0.03. On PND 8 and 10, the χ^2^ was not significant (χ^2^ = 2.58 for day 8 and χ^2^ = 1.15 for Day 8).

No significant effects or interactions were found for the 4-way ANOVA testing NOP in the Stranger condition. The effect of genotype and treatment yielded an F value <1 and the effect of sex F (1,52) = 1.48, n.s., and Day F (2,104) = 1.09 as well as all relevant interactions were not significant (Table 1). In addition, comparing the genotypes by non-parametric χ^2^ analysis did not result in significant differences between the PON1^-/-^ and PON1^+/+^ mice.

**Table 1:** Nest odor preference index compared to odor from a strange cage; [(Home odor—Stranger odor) / (Home odor + stranger odor)]. Data presented are Mean (SEM). The number of pups per group is the same as in the legend for Fig 4.

	PON1^+/+^		PON1 ^-/-^	
Day 8	Male	Female	Male	Female
Vehicle	0.094 (0.25)	0.176 (0.33)	0.509 (0.17)	0.123 (0.28)
CPF	0.258 (0.33)	0.528 (0.34)	0.113 (0.20)	0.281 (0.18)
**Day 10**				
Vehicle	0.437 (0.28)	0. 682 (0.12)	-0.002 (0.23)	0.318 (0.22)
CPF	0.548 (0.17)	0. 540 (0.19)	0.263 (0.19)	0.498 (0.17)
**Day 12**				
Vehicle	0.175 (0.13)	0.369 (0.29)	0.223 (0.20)	0.359 (0.17)
CPF	0.184 (0.27)	0.561 (0.20)	0.366 (.12)	0.281 (0.15)

In summary, PON1^-/-^ mice were delayed in developing the righting reflex but this effect was not modified by prenatal CPF exposure. CPF specifically altered the sex difference in the development of the NG reflex, resulting in higher scores in female pups compared to males. PON1^-/-^ mice also showed diminished preference for the home nest odor, an effect that was exacerbated by the CPF exposure during gestation. This suggests that the PON1-/- mice show early signs of the social deficits and the increased susceptibility to social deficits after exposure to CPF might be a prelude to the social impairments reported in adult PON1 ^-/-^ mice in Experiment 2.

## Discussion

This study describes, for the first time, a behavioral phenotype in PON1^-/-^ mice that shares features that are part of the ASD phenotype in humans, namely reduced spontaneous and learned social behavior as well as slower development of sensori-motor reflexes. The goal of the study was to determine whether PON1^-/-^ mice would be more vulnerable to the effects of gestational exposure to CPF than are PON1^+/+^ mice. Diminished social behavior in adult PON1^-/-^ pups and adult mice that had been exposed during gestation to CPF, compared to the vehicle-treated control group, lent moderate support to this hypothesis.

Pup social affiliation, assessed by the NOP test was reduced in the PON1^-/-^ mice treated with CPF. However, even in the vehicle-treated PON1^-/-^ pups’ affiliative behavior in the NOP was significantly lower than that of the PON1^+/+^ mice, suggesting that this genotype has less social affiliation than PON1^+/+^ mice. Early assessment of social deficits in pups bolsters the burgeoning research in infants focusing on early diagnosis of ASD via measures such as gaze fixation [63], auditory evoked potentials [64] or joint attention [65]. NOP was reduced in rat pups by prenatal stress to the dam [55, 66] and enhanced in male, but not female mouse pups with the Chd8^+/N2373K^ mutation [67]. We suggest that the reduced NOP might be a precursor to the diminished interest in social stimuli seen in adult mice. Early detection is an avenue worth exploring, in order to assess early remediation of social deficits [68] in rodent models. The delayed development of pup reflexes reported here in males treated with CPF and in the PON1-/- genotype mirrors the subtle neurological signs reported in children and infants who later are diagnosed with ASD. Abnormal reflexes and signs such as toe-walking, albeit neither specific or definitive, are recorded more frequently in children who are subsequently diagnosed with ASD compared to healthy controls [69–71].

Sex differences in this study can be attributed to various factors that require further investigation (reviewed in [72]. ASD is more common in males than in females [73, 74], as observed in some genetic mouse models of the disorder [66]. This suggests that males have an inherent vulnerability to the adverse effects of exposure to CPF and other OPs during gestation, [75, 76].

The major outcome of this series of experiments was the consistent impoverished social behavior in the PON1^-/-^ pups and adult mice. Social anhedonia, defined as reduced enjoyment of social contact (‘liking’), reduced anticipation of social stimuli (‘wanting’) and impaired learning for social reward are characteristic features of ASD [77, 78]. As there are no previous descriptions of the behavioral phenotype of the PON1^-/-^ mice, the findings recorded here warrant further investigation of the role of the PON1 gene, by examining heterozygotes and by exploring the contribution of maternal behavior via cross-fostering experiments.

The possibility of a general learning deficit induced in the PON1-/- mice was equivocal due to small number of mice, so that the cognitive aspects of the PON1^-/-^ behavioral phenotype remain unchartered territory. Although Cole et al. [42] did not find impaired spatial learning and contextual fear in the PON1^-/-^ mice, despite exposure to CPF oxon during gestation, that study did not compare the behavior of PON1^-/-^ mice to wild type mice, precluding the possibility of detecting a learning deficit in the PON1^-/-^ genotype.

## Conclusion

This study a) demonstrated that the PON1^-/-^ genotype may be used to further explore the mechanisms underlying ASD like behavior and b) showed that even low doses of CPF during gestation have adverse effects on the development of social behavior. The interaction between PON1 status and OP exposure and the link between PON1 and social behavior have potential practical implications. PON1 status could be modified by reduction of smoking [16–17] reduced cholesterol consumption [79]. Lipid metabolism is altered in children with autism [80]; yet the link to paraoxonase activity remains to be explored.

## Supporting information

S1 Data (7Z)
